# State of the JACMP: Thoughts on our transition and a fond farewell to Multimed

**DOI:** 10.1120/jacmp.v17i6.6751

**Published:** 2016-11-08

**Authors:** Michael D. Mills

As I write this Editorial on October 25, I realize it will be the last document submitted through the Public Knowledge Project (PKP) system and published by Multimed. Beginning October 26, we will accept submissions and resubmissions within the Wiley/Electronic Journal Press systems through the links provided in the Announcements and in the Section Editor/Author/Reviewer corresponding emails. We intend to keep the PKP system live through December 31, but it will be closed down on January 1 and replaced by the new Wiley/EJP site. Please help us by completing any reviews that you may have been asked to do. By December 31 we must not have any articles left in the PKP system.

Sometime ago, I was asked to write a support letter for Multimed to be used in a packet to prospective clients. This is what came to mind (some information is updated):

*To whom it may concern:*

*In the late summer of 2003, I began a worldwide search for a unique publishing partner. The Journal of Applied Clinical Physics (JACMP*, www.jacmp.org *) had completed four years of publication, but was in financial crisis. It was my responsibility to salvage the situation. The JACMP occupies a publication space that bridges medicine and science. We needed a publisher with special talents and skills, one that was familiar with the exacting standards of archival academic medical publication. Our high‐cost traditional publisher was not able to meet our requirements to lower costs to match our revenue. We needed a publisher able to maintain the high quality of our publication, but also able to publish on a cost‐effective basis*.

*My search led me to evaluate over two dozen potential publishing partners. My criteria for evaluation included:*

- *An established publisher with a 10‐year‐plus track record of excellence in medical publication*
- *A publisher that was willing to embrace the open‐access publication revolution*
- *A publisher that could work with open‐access, open‐source publication software tools*
- *A publisher able to provide copy editing and layout services that matched our existing publication model*
- *A publisher that offered customized services to preserve our advertising model*
- *A publisher that provided customized journal management and configuration tools*
- *A publisher that was able to provide services at a cost that matched our revenues*

*At the completion of my evaluation, Multimed stood above all other alternatives as the single publisher most able to meet our publication requirements. That was in September of 2003, so let me share with you what has happened since. Based on the number of published pages, JACMP has grown an average of 20% per year every year through 2016. Currently, the JACMP has become the world's leading clinical medical physics academic journal and enjoys an impressive Impact Factor of 1.444*.

*My friends at Multimed are highly skilled and experienced in their craft, timely in their responsiveness, gracious, and utterly professional in everything that they do. In every respect, I value my relationship with the extraordinary members of the publication team at Multimed. While the future of the JACMP was once in doubt, it is now an international archival medical journal of the highest quality. Multimed deserves substantial credit for this accomplishment. I am confident they can make your publication team as successful as we are with the JACMP*.

*Sincere Best Wishes*,

*Michael D. Mills, Ph.D., Editor‐in‐Chief, JACMP, 2004–2007, 2013‐present*

Leaving Multimed is bittersweet, as they have been an instrumental part of *JACMP*'s success. So many thanks to Jill, Amanda, Laura, Heather, Patricia, Trudi and Sharon. Together we have helped millions of cancer patients and it was a pleasure to partner with you.

## COPYRIGHT

This work is licensed under a Creative Commons Attribution 3.0 Unported License.

